# Proteomics unveil candidate biomarkers and pathogenesis of subacute thyroiditis

**DOI:** 10.1530/EC-24-0535

**Published:** 2025-02-04

**Authors:** Litong Ran, Xiufei Liu, Yongfeng Tian, Jiaran Zhu, Zhengyuan Gong, Qiao Qiao, Xin Jiang, Yuren Wang, Guojun Yang, Hongting Zheng, Yi Zheng, Hua Qu

**Affiliations:** ^1^ Department of Endocrinology, Translational Research of Diabetes Key Laboratory of Chongqing Education Commission of China, Second Affiliated Hospital of Army Medical University, Chongqing, China; ^2^ Department of Clinical Laboratory, The Second Affiliated Hospital of the Army Medical University, Chongqing, China; ^3^ The 2nd Affiliated Hospital of Guizhou University of TCM, Guizhou, China

**Keywords:** subacute thyroiditis, proteomics, biomarkers, fine-needle aspiration biopsy samples, plasma

## Abstract

**Abstract:**

Subacute thyroiditis (SAT) is an inflammatory thyroid disease characterized by neck pain, tenderness, general symptoms and thyroid dysfunction. Despite gaining new insights into the epidemiology, pathogenesis and treatment of SAT in recent years, the exact pathogenesis and determinants of its clinical progression remain unclear. Here, we profiled thyroid *in situ* protein alterations in fine-needle aspiration biopsy samples from SAT patients using proteomic analysis and uncovered 57 differentially abundant proteins. Gene ontology and KEGG enrichment analyses identified that these proteins were enriched in processes involving infection, inflammatory response and cell adhesion and junction, which likely contribute to the pathogenesis. Moreover, the top three high-abundance proteins (nicotinamide N-methyltransferase (NNMT), FTL and thymidine phosphorylase (TYMP)) were further validated in the plasma from a larger SAT cohort using an enzyme-linked immunosorbent assay. After adjusting for sex, Spearman correlation analysis showed that NNMT, FTL and TYMP levels were positively correlated with FT3, FT4, T3, T4, Tg and erythrocyte sedimentation rate and negatively correlated with thyroid-stimulating hormone. Furthermore, binary logistic regression analyses revealed that NNMT, FTL and TYMP were independent factors of SAT. We also conducted a receiver operating characteristic curve analysis to assess the diagnostic accuracy of NNMT, FTL and TYMP for SAT. The results revealed that each factor demonstrated an area under the curve score above 0.8. Thus, these high-abundance proteins can potentially serve as biomarkers for SAT diagnosis and outcome prediction. Our findings provide valuable insights into SAT biomarkers and shed light on the potential pathogenesis and therapeutic targets of SAT.

**Highlights:**

- Proteomic profiling identifies NNMT, FTL and TYMP as potential biomarkers for SAT.
- This study reveals significant enrichment of infection, inflammatory response and cell adhesion pathways in SAT pathogenesis.
- Elevated plasma NNMT, FTL and TYMP levels reflect SAT-related inflammatory and metabolic alterations.

## Introduction

Subacute thyroiditis (SAT, also known as granulomatous thyroiditis or de Quervain’s thyroiditis) was first described in 1895 by Mygind as an inflammation of the thyroid gland without abscess formation (1). Although it is a self-limiting pathology, neck, local and general symptoms and thyroid dysfunction can persist for months in untreated patients. Furthermore, the occurrence of permanent hypothyroidism was noted to be between 11 and 26% in patients with SAT. However, the pathogenesis and triggering factors of SAT are still poorly understood, and some factors, such as viral infection, change with the epidemiological characteristics of the virus (2). For example, coxsackievirus, mumps, Epstein–Barr virus and influenza virus have been previously considered to contribute to the development of SAT (2, 3, 4). During the COVID-19 pandemic, the annual SAT incidence has been reported to increase along with the reduction in other SAT-related viral infections; thus, COVID-19 is deemed as a new trigger factor for this disease (5, 6). Considering the wide variety of triggering factors and that these factors may change with the alteration of the disease spectrum, it is of great significance to explore the molecular mechanism of pathogenesis and identify potential biomarkers for SAT prevention, early diagnosis and treatment.

Most diagnostic criteria of SAT are exclusionary and are mainly based on the presence of clinical symptoms and a significant alteration in biochemical inflammation markers, including erythrocyte sedimentation rate (ESR) and/or C-reactive protein (CRP) (7, 8). Both inflammation markers lack specificity for SAT, making the exclusionary diagnosis of SAT more challenging (9). Plasma protein concentrations have been widely used as indicators of pathophysiological changes caused by various diseases (10). Here, we profiled thyroid *in situ* responses to SAT in humans by performing quantitative proteomics of fine-needle aspiration biopsy (FNAB) samples from SAT patients and revealed a number of secretory proteins altered in SAT conditions. The top three high-abundance proteins were further validated via enzyme-linked immunosorbent assay (ELISA) in plasma samples from a larger SAT cohort. These high-abundance proteins may play critical roles in the pathophysiological mechanism of SAT, and abnormal alterations of these proteins in patient plasma may serve as biomarkers for the diagnosis of SAT. Thus, combined with the thyroid *in situ* proteomics and plasma protein profiles, the current study provides valuable information on biomarkers associated with SAT, which may provide insights into the pathogenesis and potential targets for therapeutic intervention.

## Methods

### Study population and setting

**Figure 1:**
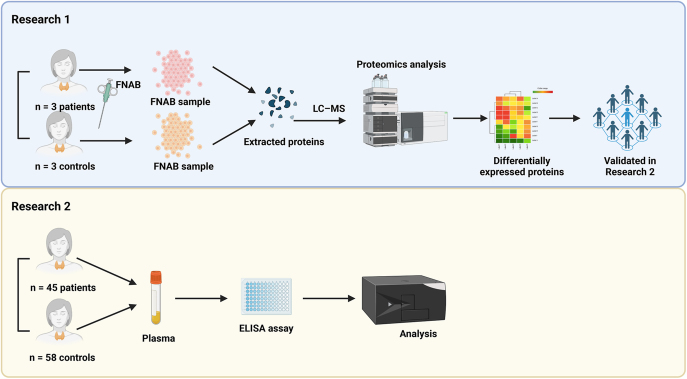
Study design. In research #1, human thyroid FNAB samples were obtained from three SAT patients and three healthy controls. Proteins were extracted from the samples and subjected to proteomic analysis. Differentially abundant proteins were analyzed using GO and KEGG enrichment analyses. In research #2, plasma samples were obtained from 45 patients with SAT and 58 healthy controls. The top three high-abundance proteins identified in research #1 were further confirmed in this larger cohort by ELISA.

The current study follows the Strengthening the Reporting of Observational Studies in Epidemiology (STROBE) reporting guidelines. The study design is illustrated in Fig. 1. In research #1, human thyroid FNAB samples were obtained from three SAT patients (whose SAT was diagnosed according to the 2016 American Thyroid Association (ATA) guidelines (1) combined with the pathological test results) and three healthy controls (confirmed by pathological test results). In research #2, plasma samples were obtained from 45 patients with SAT and 58 healthy controls. In this study, SAT was diagnosed according to the 2016 ATA guidelines (1). All samples were collected according to a protocol approved by the institutional review board of the Ethics Committee of the Second Affiliated Hospital of Army Medical University (approved protocol number 2016-056-01). All procedures were conducted in compliance with the principles of the Declaration of Helsinki, and all participants provided written informed consent.

### Clinical measures

Thyroid function tests, including thyroid-stimulating hormone (TSH), thyroxine (T4), triiodothyronine (T3), free T4 and free T3, were performed using an ARCHITECT i2000SR immunoassay analyzer. Thyroid antibodies, including anti-thyroid peroxidase antibodies (anti-TPO antibodies, TPOAb), thyrotropin receptor antibodies (TRAb), thyroglobulin antibodies (TgAb) and thyroglobulin (Tg), were assessed using the Mindray Medical CL-6000i chemiluminescence immunoassay system. The ESR was measured using a Tianhai ESR-2040 dynamic ESR analyzer. CRP and white blood cell (WBC) counts were analyzed using an automated Sysmex XN-9000 hematology system.

### Proteomic analysis of FNAB samples

The lysis buffer (8 mol/L urea and 1% protease inhibitor cocktail) was added to the FNAB samples and sonicated three times on ice using a high-intensity ultrasonic processor (Scientz, China). The remaining debris was removed by centrifugation at 12,000 *g* for 10 min at 4°C, and the supernatant was collected. Protein concentrations were assessed using a BCA kit, according to the manufacturer’s instructions. The collected proteins were digested with trypsin. The protein solution underwent a 30-min reduction at 56°C with 5 mM dithiothreitol and was subsequently alkylated in darkness at room temperature with 11 mM iodoacetamide for 15 min. To achieve a urea concentration of less than 2 M, the protein sample was diluted with 100 mM TEAB. Trypsin was used for two digestion phases: an initial overnight digestion at a trypsin-to-protein mass ratio of 1:50, followed by a second 4-h digestion at a trypsin-to-protein mass ratio of 1:100. LC–MS/MS analysis was conducted by PTM Bio (China) using the EASY-nLC 1200 UPLC system (Thermo Fisher Scientific, USA) and Q Exactive HF-X (Thermo Fisher Scientific) with a nano-electrospray ion source. Tryptic peptides were dissolved in solvent A (0.1% formic acid and 2% acetonitrile in water) and loaded onto a home-made reversed-phase column (25 cm × 75 μm i.d.). Peptide separation was carried out with a gradient from 5 to 25% solvent B (0.1% formic acid in 90% acetonitrile) over 60 min, followed by 25–35% over 22 min and then from 35 to 80% over 4 min, with a final 4-min hold at 80%, at a flow rate of 450 nL/min using an EASY-nLC 1200 UPLC system (Thermo Fisher). Separated peptides were analyzed on a Q Exactive HF-X mass spectrometer (Thermo Fisher) with a 2.0 kV nano-electrospray ion source. Full MS scans were performed at a resolution of 60,000 (350–1600 *m*/*z*), and the top 20 most abundant precursors were selected for MS/MS with 30-s dynamic exclusion. HCD fragmentation was performed with a normalized collision energy of 28%, and fragments were detected in the Orbitrap at 30,000 resolution. The first fixed mass was set to 100 *m*/*z*, with an automatic gain control target of 1E5, an intensity threshold of 3.3E4 and a maximum injection time of 60 ms. The MS/MS data were processed using MaxQuant (v.1.6.15.0), with database searches against the human SwissProt database (v.2023_01, 20,422 entries) and a reverse decoy database. Trypsin/P was selected as the enzyme, allowing up to two missed cleavages. Mass tolerances were set to 20 ppm for precursor ions in the first search and 5 ppm for the main search, and 0.02 Da for fragment ions. Carbamidomethylation of cysteine was set as a fixed modification, and acetylation at the N-terminus and oxidation of methionine were set as variable modifications, with an FDR threshold of <1%.

### Potential SAT biomarker measures

The top three high-abundance proteins identified in the proteomic analysis of FNAB samples were further validated in plasma samples from research #2. Plasma concentrations of nicotinamide N-methyltransferase (NNMT), thymidine phosphorylase (TYMP) and FTL were measured using ELISA kits, following the manufacturer’s instructions. All samples were assayed in duplicate, and in cases where there was a >15% difference between duplicates, the assay was repeated. The detection range for NNMT was 5–160 U/L; for TYMP, it was 1.56–100 ng/mL; and for FTL, it was 0.39–25 ng/mL. The intra-assay coefficient of variance (CV) was 10%, and the inter-assay CV was 12%. No significant cross-reactivity or interference was observed during analysis.

### Statistical analysis

Data analyses were performed using the SPSS software (IBM, USA, version 19.0) and GraphPad Prism (GraphPad Software Inc., USA, version 8.0). All data are presented as the mean ± standard deviation (SD), unless otherwise specified. The normal distribution of the data was assessed using the Kolmogorov–Smirnov test, and variables exhibiting a skewed distribution were logarithmically transformed to achieve normality before statistical analysis. Group comparisons were made using Student’s *t*-test. Bivariate correlation analysis was performed using Spearman’s correlation coefficient, a nonparametric method robust to outliers, after identifying and excluding data points with standardized residuals exceeding an absolute value of 3. To adjust for factors that might influence the association between potential biomarkers and SAT, multivariate logistic regression analysis was performed. Binary logistic regression analyses were performed to evaluate the association between potential biomarkers and SAT. In these analyses, a positive SAT diagnosis was defined as the binary-dependent variable, whereas NNMT, FTL and TYMP were included as independent variables. Statistical significance was set at *P* < 0.05.

## Results

### General characteristics

The general characteristics of all participants are presented in Tables 1 and 2. The study pool comprised 40 men and 69 women. In research #1, the average age was 48.00 ± 10.54 years in the SAT group and 47.67 ± 11.68 years in the control group (Table 1). Liver and renal functions, including ALT, AST, BUN, Cr and eGFR, were comparable between these two groups, and all were within the normal range (Table 1). Similarly, thyroid antibodies, including TPOAb, TgAb and TRAb, showed no difference between the groups, and all remained within the normal range. While Tg showed an increased trend in the SAT group, thyroid functions, including FT3, FT4, TT3 and TT4, were higher (*P* > 0.05) and TSH levels were significantly lower (*P* < 0.05) in SAT patients when compared with controls (Table 1). Inflammatory indicators, such as ESR, hsCRP and WBC, were significantly higher in the SAT group than in the control group (Table 1). Moreover, ultrasound descriptions in the SAT group were focal or multifocal lesions with poorly defined, heterogeneous and hypoechoic echogenicity. Importantly, FNAB samples from patients with SAT exhibited distinct cytological features, including follicular epithelial cells displaying degenerative changes, abundant thick colloids, numerous multinucleated giant cells and an influx of inflammatory cells, predominantly lymphocytes and macrophages. These findings align with key diagnostic characteristics as reported in previous studies (1, 9, 11).

**Table 1:** General characteristics of participants in research #1.

Clinical characteristics	SAT	Controls	*P* value
N (M/F)	3 (0/3)	3 (0/3)	-
Age (years)	48.00 ± 10.54	47.67 ± 11.68	0.97
ALT (IU/L)	22.60 ± 8.88	26.27 ± 4.41	0.56
AST (IU/L)	20.13 ± 7.05	26.90 ± 10.89	0.42
eGFR (mL/min/L)	112.33 ± 16.01	110.33 ± 3.51	0.84
Cr (μmol/L)	48.57 ± 10.93	52.40 ± 6.61	0.63
BUN (mmol/L)	3.73 ± 0.75	4.75 ± 0.55	0.13
UA (μmol/L)	283.43 ± 90.84	277.40 ± 39.83	0.92
FT3 (pmol/L)	8.10 ± 3.66	4.01 ± 0.58	0.13
FT4 (pmol/L)	23.05 ± 10.09	12.62 ± 0.91	0.15
TSH (mIU/L)	0.02 ± 0.02	1.73 ± 0.61	0.01
T3 (nmol/L)	3.04 ± 1.10	1.27 ± 0.20	0.05
T4 (nmol/L)	171.01 ± 58.93	88.71 ± 1.46	0.07
Tg (μg/L)	190.60 ± 269.21	22.87 ± 25.54	0.34
TgAb (IU/mL)	102.49 ± 142.73	19.13 ± 6.99	0.37
TPOAb (IU/mL)	5.91 ± 6.33	22.96 ± 35.56	0.46
TRAb (IU/L)	2.00 ± 1.11	1.13 ± 0.35	0.26
ESR (mm/h)	39.67 ± 14.57	11.00 ± 3.61	0.03
CRP (mg/L)	42.60 ± 21.25	2.27 ± 0.59	0.03
WBC (10^9^/L)	9.58 ± 1.54	4.79 ± 1.07	0.01

SAT, subacute thyroiditis; TSH, thyroid-stimulating hormone; T3, triiodothyronine; T4, thyroxine; Tg, thyroglobulin; TgAb, thyroglobulin antibodies; TRAb, thyrotropin receptor antibodies; ESR, erythrocyte sedimentation rate; CRP, C-reactive protein; WBC, white blood cell.

**Table 2:** General characteristics of participants in research #2.

Clinical characteristics	SAT	Controls	*P* value
N (M/F)	45 (12/33)	58 (28/30)	(0.023)
Age (years)	45.09 ± 9.69	48.06 ± 12.09	0.187
ALT (IU/L)	40.31 ± 33.59	24.56 ± 17.47	0.013
AST (IU/L)	23.83 ± 14.48	19.52 ± 12.38	0.216
BUN (mmol/L)	5.64 ± 2.06	5.86 ± 2.01	0.747
Cr (μmol/L)	60.90 ± 13.10	64.75 ± 17.90	0.505
eGFR (mL/min/L)	105.91 ± 11.71	104.79 ± 17.86	0.844
FT3 (pmol/L)	9.51 ± 6.78	4.29 ± 0.54	<0.001
FT4 (pmol/L)	25.36 ± 14.19	12.33 ± 1.36	<0.001
T3 (nmol/L)	2.90 ± 1.50	1.54 ± 1.43	<0.001
T4 (nmol/L)	178.15 ± 77.64	92.07 ± 14.56	<0.001
TSH (mIU/L)	0.66 ± 1.78	1.37 ± 0.79	0.010
TgAb (IU/mL)	85.22 ± 172.3	43.66 ± 115.59	0.172
TPOAb (IU/mL)	35.69 ± 161.59	47.77 ± 183.96	0.745
TRAb (IU/L)	2.03 ± 0.84	1.36 ± 0.68	0.099
Tg (μg/L)	128.74 ± 132.62	21.24 ± 69.61	<0.001
ESR (mm/h)	38.64 ± 27.53	3.65 ± 14.92	<0.001
CRP (mg/L)	14.77 ± 26.71	3.38 ± 15.59	0.010
WBC (10^9^/L)	7.20 ± 2.18	6.07 ± 2.05	0.015

SAT, subacute thyroiditis; T3, triiodothyronine; T4, thyroxine; TSH, thyroid-stimulating hormone; TgAb, thyroglobulin antibodies; TRAb, thyrotropin receptor antibodies; Tg, thyroglobulin; ESR, erythrocyte sedimentation rate; CRP, C-reactive protein; WBC, white blood cell.

In research #2, the average age was 45.09 ± 9.69 years in SAT group and 48.06 ± 12.09 years in controls (Table 2). ALT levels were significantly higher in patients with SAT than in controls. Other liver and renal functions, including AST, BUN, Cr and eGFR, were comparable between the two groups, and all were within the normal range (Table 2). Thyroid functions, including FT3, FT4, TT3 and TT4, were significantly higher (*P* < 0.05), and TSH levels were significantly lower (*P* < 0.05) in SAT patients than in controls. Thyroid antibodies (TPOAb, TgAb and TRAb) showed an increasing trend in SAT patients (*P* > 0.05), whereas Tg was markedly elevated in these patients (*P* < 0.05). Inflammatory indicators, such as ESR, hsCRP and WBC, were significantly higher in the SAT group than in the control group (Table 2).

### Proteomic profiling of FNAB samples from SAT patients and control volunteers

**Figure 2:**
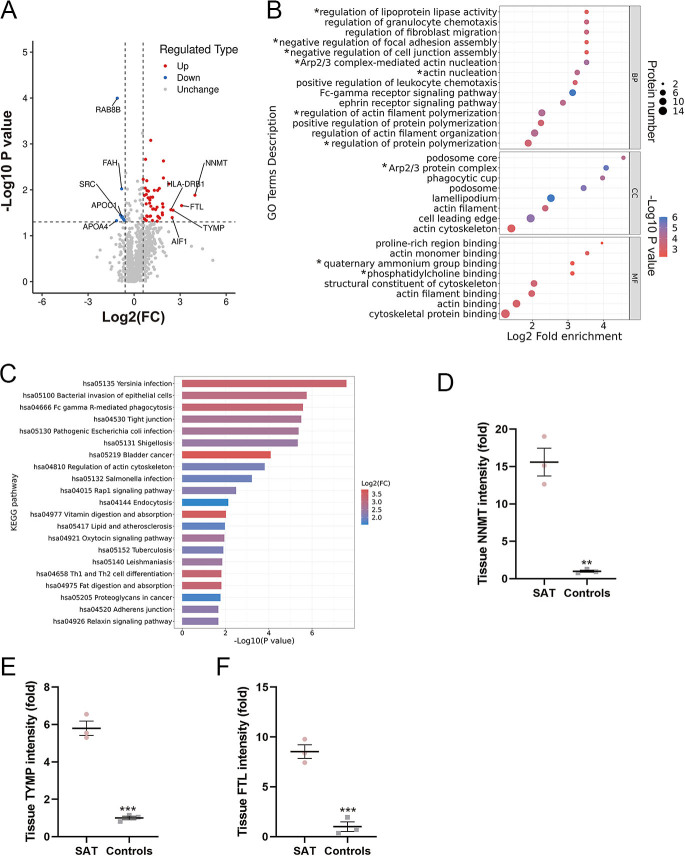
Proteomic profiling of FNAB samples from SAT patients and control volunteers. (A) Volcano plots showing the differences in protein abundance between SAT patients and control subjects. (B) GO enrichment analysis of differentially abundant proteins in FNAB samples from SAT patients versus control subjects (this result is based on a small number of proteins, *n* = 58); therefore, GO terms derived from the same set of proteins are marked with an asterisk (*). (C) KEGG pathway enrichment analysis of differentially abundant proteins in FNAB samples from SAT patients versus controls. FC, fold-change of differentially abundant proteins in SAT patients versus control subjects. (D, E, F) The intensity of tissue NNMT, TYMP and FTL expression in SAT patients compared to that in controls. Data are presented as mean ± SEM. **P < 0.01, ***P < 0.001.

From research #1, a total of 6393 peptides were collected and 1335 proteins were identified in FNAB samples from SAT patients and controls (Supplementary Table 1 (see section on Supplementary materials given at the end of the article)). By setting the fold-change in abundance as >1.5 and *P* < 0.05, 57 differentially abundant proteins were identified (49 high abundance and 8 low abundance, Fig. 2A and Supplementary Table 2). These proteins were then subjected to gene ontology (GO) (12) and Kyoto Encyclopedia of Genes and Genomes (KEGG) pathway (13) enrichment analyses. GO terms were highly enriched in processes involved in lipoprotein lipase activity, granulocyte chemotaxis, fibroblast migration, focal adhesion and cell junctions (Fig. 2B). KEGG pathways were enriched in bacterial infection, bacterial invasion, macrophage Fcγ receptor (FcγR)-mediated phagocytosis and tight junctions. These results indicate that infection, inflammatory response and cell adhesion and junction might be the most altered processes in patients with SAT (Fig. 2C). In addition, proteomic data suggested that NNMT, ferritin light chain (FTL) and TYMP were the most differentially abundant proteins in SAT patients when compared with controls (Fig. 2D, E, F).

### Plasma concentrations of NNMT, FTL and TYMP were markedly increased in SAT patients

**Figure 3:**
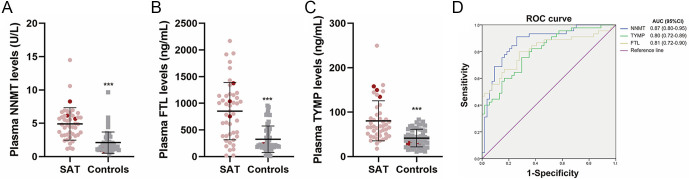
Plasma NNMT, FTL and TYMP concentrations and diagnostic efficiency for SAT. (A, B C) Plasma was collected from SAT patients and controls enrolled in research #2; NNMT, FTL and TYMP concentrations were detected using ELISA. The levels of NNMT, TYMP and FTL in SAT patients from whom the FNAB samples were obtained are marked in red. Detailed data are shown in Supplementary Table 3. (D) ROC curve showing the predictive accuracy of the indicated factors for SAT. Data are presented as mean ± SD. ***P < 0.001.

**Figure 4:**
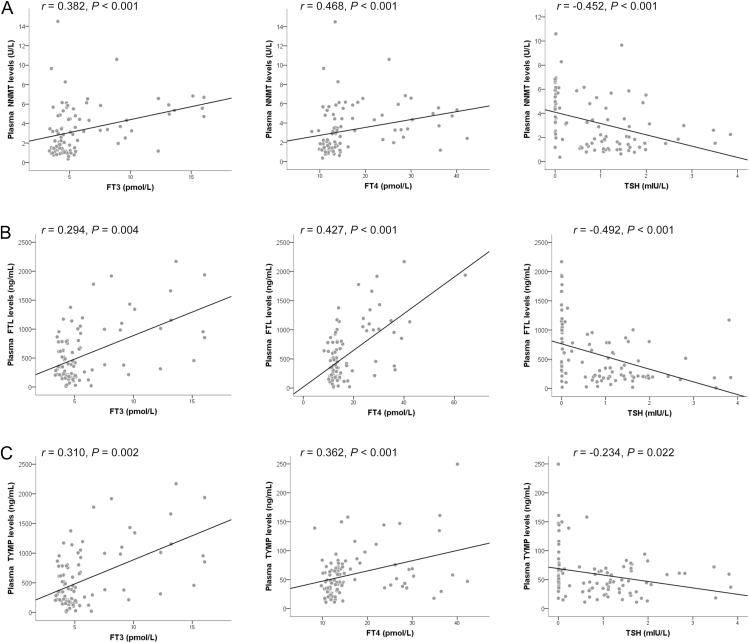
Correlation between plasma NNMT, FTL and TYMP concentrations and thyroid function indicators after outlier removal. (A, B, C) Plasma was collected from SAT patients and controls enrolled in research #2; NNMT, FTL and TYMP concentrations were detected using ELISA. Scatter plots showing the correlation between plasma protein levels (NNMT, FTL and TYMP) and thyroid function indicators (FT3, FT4 and TSH) after excluding outliers (standardized residuals with absolute values >3). Spearman’s correlation coefficient was used for robust analysis.

To identify potential biomarkers for SAT, the top three most differentially abundant proteins, which have been reported as secretory proteins by previous studies, were further evaluated using another SAT cohort (research #2). Consistently, the plasma concentrations of NNMT, FTL and TYMP were significantly elevated in SAT patients compared to those in healthy subjects (Fig. 3A, B, C). Spearman correlation analysis showed that NNMT, FTL and TYMP levels were positively correlated with FT3, FT4, T3, T4, Tg and ESR and negatively correlated with TSH (Table 3 and Fig. 4). Moreover, FTL levels were positively correlated with TgAb and TPOAb levels and NNMT levels were positively correlated with WBC count (Table 3). Furthermore, by setting SAT-positive diagnosis as a binary-dependent variable and NNMT, FTL and TYMP as independent variables, binary logistic regression analyses revealed that NNMT, FTL and TYMP were independent factors for SAT (OR = 1.78, 1.00 and 1.04, 95% CI = 1.24–2.54, 1.002–1.005, 1.01–1.07, respectively). We conducted a receiver operating characteristic (ROC) curve analysis to assess the diagnostic accuracy of NNMT, FTL and TYMP for SAT. The results revealed that each factor demonstrated an area under the curve (AUC) score above 0.8 (Fig. 3D), indicating excellent discriminatory ability for identifying SAT.

**Table 3:** Spearman correlation analysis of NNMT, FTL and TYMP with general and clinical indicators adjusted by gender.

	NNMT		FTL		TYMP	
	*r*	*P*	*r*	*P*	*r*	*P*
NNMT	-	-	0.185	0.067	0.402	<0.001
FTL	0.402	<0.001	-	-	0.144	0.155
TYMP	0.185	0.067	0.144	0.155	-	-
Age	−0.072	0.482	0.017	0.870	0.105	0.299
ALT	−0.018	0.880	0.203	0.094	−0.119	0.329
AST	−0.185	0.128	−0.007	0.951	−0.094	0.443
eGFR	−0.005	0.969	−0.035	0.790	−0.142	0.284
Cr	0.023	0.865	0.194	0.141	0.133	0.315
BUN	−0.101	0.447	−0.003	0.983	0.011	0.934
FT3	0.294	0.003	0.214	0.035	0.278	0.006
FT4	0.408	<0.001	0.321	0.001	0.343	0.001
TSH	−0.365	<0.001	−0.333	0.001	−0.234	0.047
T3	0.233	0.021	0.307	0.002	0.206	0.045
T4	0.344	0.001	0.291	0.004	0.273	0.007
Tg	0.216	0.049	0.228	0.044	0.227	0.031
TgAb	−0.141	0.183	0.218	0.038	−0.173	0.100
TPOAb	0.010	0.924	0.227	0.031	−0.053	0.618
TRAB	−0.032	0.845	0.235	0.139	−0.063	0.697
ESR	0.431	<0.001	0.306	0.002	0.279	0.005
CRP	0.169	0.096	0.052	0.610	0.152	0.136
WBC	0.210	0.047	0.182	0.087	0.128	0.228

NNMT, nicotinamide N-methyltransferase; TYMP, thymidine phosphorylase; TSH, thyroid-stimulating hormone; T3, triiodothyronine; T4, thyroxine; Tg, thyroglobulin; TgAb, thyroglobulin antibodies; ESR, erythrocyte sedimentation rate; CRP, C-reactive protein; WBC, white blood cell.

## Discussion

The current study recruited two cohorts of patients with SAT and control volunteers. In research #1, all participants underwent the FNAB test, and the samples were analyzed using proteomic-based approaches. In total, 57 differentially abundant proteins were identified, and GO and KEGG enrichment analyses suggested that these proteins were enriched in processes involving infection, inflammatory response and cell adhesion and junction. Moreover, the top three high-abundance proteins were identified as NNMT, FTL and TYMP and were further confirmed in a larger SAT cohort, that is, research #2. The three high-abundance proteins were secretory proteins, and their plasma concentrations were higher in SAT patients than in controls, which was consistent with our proteomic findings. Further correlation analysis showed that the plasma levels of NNMT, FTL and TYMP were significantly associated with thyroid function, Tg and ESR. Further binary logistic regression analyses suggested that NNMT, FTL and TYMP were independent factors for SAT, and ROC curve analysis indicated that these factors had excellent discriminatory ability in identifying SAT. The alterations in these proteins not only offer valuable insights into the pathogenesis of SAT but also demonstrate promising potential for further development as clinical biomarkers to closely monitor and evaluate progression of SAT.

SAT is generally considered a self-limiting post-viral inflammatory disorder of the thyroid gland. Its diagnosis is primarily clinical, relying on the identification of an enlarged, tender thyroid gland in patients with a relevant clinical history, ultrasonography features and laboratory evidence of thyrotoxicosis. In addition, ESR and hsCRP are recommended as biomarkers not only for diagnosis but also for predicting disease progression and outcome. However, neither a technetium scan nor iodine uptake findings were mandatory diagnostic criteria for SAT. Recently, using isotope scanning with technetium, Zornitzki *et al.* have reported that only about two-thirds of patients suffered from sore or painful throat, and about half of these patients experienced fever and showed increased inflammatory markers, in contrast to previous data in which painful throat or fever was reported in most patients (14). These findings suggest that there remains a diagnostic challenge for SAT and the use of different diagnostic criteria may further diversify the clinical picture of SAT. Using FNAB samples, our current proteomic analysis profiled the protein expression alterations potentially induced by SAT. After verification in a larger cohort, our study suggests that the top three high-abundance proteins, NNMT, FTL and TYMP, may serve as new biomarkers for SAT diagnosis. Moreover, ROC curve analysis in our study demonstrated that NNMT, FTL and TYMP achieved AUC values above 0.8, indicating excellent diagnostic performance. Thus, these biomarkers showed a potential ability to distinguish patients with SAT from healthy controls, which aligns with the observed correlations between these proteins and thyroid function indicators. Compared to the current diagnostic criteria for SAT, such as relying heavily on clinical symptoms (e.g., neck pain and tenderness), and nonspecific inflammation markers, such as ESR and CRP, our biomarkers provide several potential advantages. Unlike ESR and CRP, which may be elevated under various inflammatory conditions and lack specificity for SAT, NNMT, FTL and TYMP are specific to SAT’s underlying pathophysiology of SAT, as identified through proteomic profiling and pathway analysis. These biomarkers may improve diagnostic precision, particularly in atypical cases in which clinical symptoms and standard markers are inconclusive. However, the identification of new biomarkers also presents challenges. For instance, validation in larger and more diverse cohorts is necessary to confirm their generalizability. Moreover, the cost and accessibility of ELISA-based detection methods might limit their immediate clinical applicability compared to readily available and inexpensive ESR and CRP tests. In summary, while NNMT, FTL and TYMP offer promising diagnostic specificity and mechanistic insights, further studies are required to optimize their integration into clinical practice and to compare their utility against established markers in broader, real-world populations.

Regarding the pathology of SAT, genetic background and the occurrence of certain types of human leukocyte antigens (HLAs) have been reported to be associated with SAT susceptibility. In 1975, Nyulassy *et al.* first reported that the frequency of HLA-B*35 increased in patients with SAT (15). Subsequently, several HLA loci were identified as markers of genetic susceptibility and clinical course to SAT, such as HLA-B*18:01, HLA-DRB1*01, HLA-DRB1*15:01 and HLA-B*07:02 (16). However, their results were highly inconsistent, and a causal relationship between HLA alleles and SAT has not yet been established. In addition, viral infection is generally considered a triggering factor for SAT in genetically predisposed individuals (2). In recent years, the SARS-CoV-2 pandemic has spread worldwide and has been identified as a triggering factor for SAT. The simultaneous presence of SAT has been reported in up to 20% of hospitalized patients with SARS-CoV-2 infection. In these patients, the incidence of painless SAT was significantly higher than that reported for other triggering viruses, which was thought to be related to SARS-CoV-2-induced lymphocytopenia. However, the exact pathology of SAT remains unclear. In the current study, GO and KEGG pathway enrichment analyses implied that lipoprotein lipase activity, granulocyte chemotaxis, fibroblast migration, focal adhesion, bacterial infection and invasion, FcγR-mediated phagocytosis and tight junctions may also contribute to the development of SAT, and further studies are required to assess these possibilities.

ESR and hsCRP are generally considered as biomarkers for the prognostic outcome of SAT. The specificity of these indicators is low and inflammation in various parts of the body may lead to their elevation. Our study identified the three most abundant proteins: NNMT, FTL and TYMP. NNMT plays a crucial role in nicotinamide metabolism. It catalyzes the transfer of a methyl group from S-adenosylmethionine to nicotinamide, producing S-adenosylhomocysteine and 1-methylnicotinamide. This reaction is a key step in the methylation pathway, which is essential for various cellular processes, including DNA methylation, gene expression and the regulation of cellular metabolism. Previous studies have investigated the relationship between NNMT and thyroid disease. One study found that NNMT expression was increased in the thyroid glands of patients with Graves’ disease. Another study found that NNMT expression was increased in the thyroid glands of patients with thyroid cancer (17). These findings suggest that NNMT is involved in the pathogenesis of thyroid diseases. FTL is one of the two subunits of ferritin, a protein complex that stores iron in a nontoxic and bioavailable form, which plays a crucial role in iron metabolism and storage (18). Although there has been limited direct research on the specific role of FTL in thyroid disease, previous studies have suggested a potential link between ferritin levels and thyroid cancer. Thus, it is possible that ferritin, including FTL, may be involved in the pathogenesis or progression of thyroid cancer, but more researches are needed to clarify this relationship. TYMP plays a crucial role in thymidine metabolism. It catalyzes the reversible phosphorylation of thymidine to thymine and 2-deoxy-D-ribose-1-phosphate. This reaction is a key step in the salvage pathway of DNA synthesis, which allows cells to recycle thymidine and other nucleosides to maintain DNA integrity and repair damaged DNA (19). Regarding the relationship between TYMP and thyroid disease, a previous study suggested that TYMP is a candidate biomarker for thyroid follicular adenomas (20). However, similar to FTL, further studies are required to confirm these results and their association with other thyroid diseases. The interplay among these high-abundance proteins suggests a coordinated contribution to SAT pathogenesis. NNMT and FTL may work synergistically to amplify oxidative stress and inflammatory damage, whereas TYMP enhances immune cell recruitment and vascular remodeling. Together, these processes may exacerbate the inflammatory cascade and drive clinical manifestations of SAT. Although our study provides significant insights into the association of these high-abundance proteins with SAT, it is important to acknowledge that the lack of functional experiments limits the direct attribution of causality. Further studies are warranted to elucidate the precise molecular mechanisms by which these proteins influence thyroid inflammation and their potential as therapeutic targets.

## Limitations

This study had some limitations. First, the sample size of the discovery cohort was small. Therefore, the identified biomarker candidates may represent only a subset of SAT-specific molecules. Owing to the cross-sectional nature of the study, no causal relationship between these three high-abundance proteins and SAT could be inferred. We cannot completely exclude the influence of liver and kidney function on protein synthesis and excretion and its impact on the results of the correlation analysis.

## Conclusions

The current study, which combined proteomic data from FNAB samples and plasma protein expression information from two SAT cohorts, revealed that SAT-related differentially abundant proteins were enriched in lipoprotein lipase activity, granulocyte chemotaxis, fibroblast migration, focal adhesion, bacterial infection and invasion, FcγR-mediated phagocytosis and tight junctions. These findings suggest that these pathways or processes may underlie the development of SAT. Moreover, the three most abundant proteins, NNMT, FTL and TYMP, identified by proteomics, were found to be increased in the plasma samples from a larger SAT cohort and might be independent factors for SAT by binary logistic regression analyses. Furthermore, ROC curve analysis indicated that these factors had excellent discriminatory ability in identifying SAT. Therefore, these proteins may serve as candidate biomarkers for SAT diagnosis and treatment.

## Supplementary materials









## Declaration of interest

The authors declare that there is no conflict of interest that could be perceived as prejudicing the impartiality of this work.

## Funding

This work was supported by the National Natural Science Foundation of China (Nos. 82230025, 82470897, 82470912, U24A20673 and 82270882), the Chongqing Young and Middle-aged Medical High-end Talent Program and the Chongqing Municipal Health Commission Medical Research Project (2022C091).

## Author contribution statement

Hua Qu, Xiufei Liu, Yongfeng Tian, Qiao, Zhengyuan Gong and Xin Jiang designed and conducted experiments. Jiaran Zhu and Litong Ran acquired the data and performed statistical analysis. Yuren Wang and Guojun Yang analyzed and interpreted the data. Hua Qua and Litong Ran drafted the manuscript. Hua Qu, Xiufei Liu and Yongfeng Tian critically revised the manuscript for important intellectual content. Hua Qu, Yi Zheng and Hongting Zheng were the guarantor of this work and, as such, had full access to all the data in the study and took responsibility for the integrity of the data and the accuracy of the data analysis.
